# Prediction of acute coronary syndrome within 3 years using radiomics signature of pericoronary adipose tissue based on coronary computed tomography angiography

**DOI:** 10.1007/s00330-021-08109-z

**Published:** 2021-08-25

**Authors:** Jin Shang, Shaowei Ma, Yan Guo, Linlin Yang, Qian Zhang, Fuchun Xie, Yue Ma, Quanmei Ma, Yuxue Dang, Ke Zhou, Ting Liu, Jinzhu Yang, Yang Hou

**Affiliations:** 1 Department of Radiology, Shengjing Hospital of China Medical University, Shenyang, 110004 China; 2 GE Healthcare, Shanghai, China; 3 Department of Radiology, First Affiliated Hospital of China Medical University, Shenyang, 110001 China; 4 Key Laboratory of Intelligent Computing in Medical Image (MIIC), Ministry of Education, Northeastern University, Shenyang, 110169 Liaoning China

**Keywords:** Computed tomography angiography, Adipose tissue, Inflammation, Acute coronary syndrome, Radiomics

## Abstract

**Objectives:**

To evaluate whether radiomics signature of pericoronary adipose tissue (PCAT) based on coronary computed tomography angiography (CCTA) could improve the prediction of future acute coronary syndrome (ACS) within 3 years.

**Methods:**

We designed a retrospective case-control study that patients with ACS (*n* = 90) were well matched to patients with no cardiac events (*n* = 1496) during 3 years follow-up, then which were randomly divided into training and test datasets with a ratio of 3:1. A total of 107 radiomics features were extracted from PCAT surrounding lesions and 14 conventional plaque characteristics were analyzed. Radiomics score, plaque score, and integrated score were respectively calculated via a linear combination of the selected features, and their performance was evaluated with discrimination, calibration, and clinical application.

**Results:**

Radiomics score achieved superior performance in identifying patients with future ACS within 3 years in both training and test datasets (AUC = 0.826, 0.811) compared with plaque score (AUC = 0.699, 0.640), with a significant difference of AUC between two scores in the training dataset (*p* = 0.009); while the improvement of integrated score discriminating capability (AUC = 0.838, 0.826) was non-significant. The calibration curves of three predictive models demonstrated a good fitness respectively (all *p* > 0.05). Decision curve analysis suggested that integrated score added more clinical benefit than plaque score. Stratified analysis revealed that the performance of three predictive models was not affected by tube voltage, CT version, different sites of hospital.

**Conclusion:**

CCTA-based radiomics signature of PCAT could have the potential to predict the occurrence of subsequent ACS. Radiomics-based integrated score significantly outperformed plaque score in identifying future ACS within 3 years.

**Key Points:**

*• Plaque score based on conventional plaque characteristics had certain limitations in the prediction of ACS.*

*• Radiomics signature of PCAT surrounding plaques could have the potential to improve the predictive ability of subsequent ACS.*

*• Radiomics-based integrated score significantly outperformed plaque score in the identification of future ACS within 3 years.*

**Supplementary Information:**

The online version contains supplementary material available at 10.1007/s00330-021-08109-z.

## Introduction

Acute coronary syndrome (ACS) can be often the first manifestation of coronary artery disease (CAD) and the main cause of death in the majority of the world’s population [1, 2]. As a widely used non-invasive imaging modality, coronary computed tomography angiography (CCTA) has shown its clinical value by enabling robust coronary plaque characterization and quantification [3, 4], especially for the identification of adverse plaque characteristics (APC) [5]. As plaque rupture is a complicated biomechanical process, whether the clinical outcome of vulnerable plaques developed into ACS may be affected by several factors. Among them, vascular inflammation is recognized as a key factor to both plaque formation and rupture, resulting in the occurrence of subsequent ACS [6]. A recent review of randomized controlled trials suggested that reduction of inflammation with colchicine on patients with CAD could lower the incidence of ACS [7]. Therefore, a comprehensive evaluation combining plaque characteristics with vascular inflammation may further enhance the prediction of ACS.

It has been established that there is a constant bidirectional manner between the vascular wall and pericoronary adipose tissue (PCAT); pro-inflammatory factors released from the inflamed vascular wall spread to PCAT in a paracrine manner to inhibit preadipocytes differentiation and lipid accumulation [8–10]. Antonopoulos AS et al proposed a novel imaging biomarker, fat attenuation index (FAI), that captured the CT attenuation changes of PCAT and further revealed changes in PCAT composition induced by vascular inflammation [10]. Recent clinical studies indicated that PCAT attenuation measured from CCTA may contribute to identify high-risk plaque (HRP) progression and improve prediction for adverse cardiac events [11, 12]. Nevertheless, the changes in PCAT composition were not only related to vascular inflammation but also to dysfunctional adipose tissue remodeling characterized by fibrosis and vascularity [13, 14]. Thus, this approach only relying on CT attenuation to reflect changes in PCAT composition without considering complicated spatial relationship among voxels might lead to certain overlaps between pathologies.

Radiomics refers to the process of converting digital medical images into mineable higher dimensional data, the high-throughput extraction of quantitative image features for providing clinical-decision support [15, 16]. Radiomics was originally applied in oncology. Notably, it has been gradually applied to the coronary lesions and myocardium [17–21]. Oikonomou EK et al [22] primarily proposed that a novel radiotranscriptomic signature of PCAT could detect additional disease-related changes in PCAT composition; meanwhile, their machine learning–powered radiomics analysis of PCAT could lead to a significant improvement of cardiac risk prediction. A recent report [23] based on CCTA-based radiomics characterization of PCAT surrounding coronary plaques found that there was a distinct PCAT radiomics phenotype between patients with acute MI and patients with stable or no CAD, yet the predictive value of PCAT radiomics surrounding plaques for future adverse cardiac events has not been mentioned. Therefore, our study aimed to develop a CCTA-based radiomics signature of PCAT surrounding coronary lesions to identify patients with future ACS within 3 years.

## Material and methods

### Study design and population

**Fig. 1:**
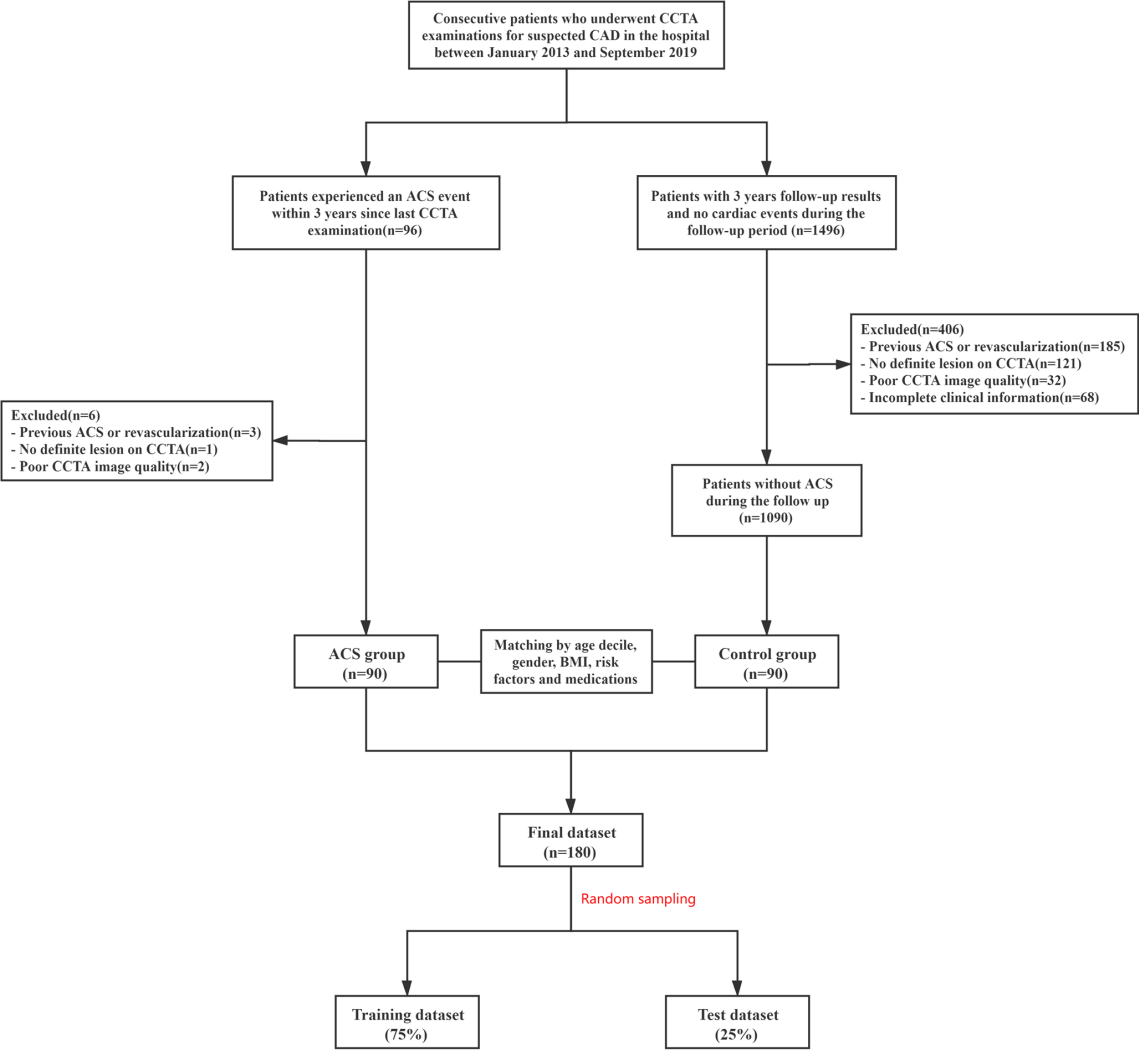
A flowchart of patient recruitment and study design. CCTA, coronary computed tomography angiography; CAD, coronary artery disease; ACS, acute coronary syndrome; BMI, body mass index

The study population was retrospectively enrolled consecutive patients who underwent CCTA examinations for suspected CAD from two different sites of our hospital between January 2013 and September 2019. We included patients who experienced an ACS event within 3 years since the last CCTA examination and had a culprit lesion identified on invasive coronary angiography. Patients with ACS were well matched (according to age decile, gender, body mass index, traditional cardiovascular risk factors, and baseline medications) to patients without adverse cardiac events during 3 years follow-up. A flowchart of patient recruitment and study design is presented in Fig. 1. This retrospective study design was approved by the local institutional review board (No. 2021PS010K), and no informed consent was required.

### CCTA acquisition

All the CCTA scanning was performed on either a 256-slice CT scanner or a dual-layer SDCT. Further details regarding the CCTA protocols from two different sites of our hospital were provided in Supplementary Materials.

### Coronary plaque analysis

Quantitative and qualitative analysis of coronary plaques (Supplementary Materials) were performed in culprit lesions of patients with ACS and highest-grade stenosis lesions of control patients by an independent observer who was blinded to the clinical data and CCTA results with semi-automated software (IntelliSpace Portal, Philips Healthcare). Lumen and vessel contours were manually adjusted if necessary. Finally, a total of 14 conventional plaque characteristics were obtained.

### PCAT segmentation and radiomics feature extraction

Given that recent studies have shown that PCAT surrounding coronary plaques have the potential to become a sensitive imaging marker of plaque vulnerability [23–25], we performed PCAT segmentation around culprit lesions in patients with ACS. Since there was no culprit lesion in the control group, we chose the highest-grade stenosis nonculprit lesion of each patient for PCAT radiomics phenotyping [23]. PCAT was defined as all the voxels between −190 and −30 HU range located within a radial distance from the outer vessel wall equal to the average diameter of the target vessel. The detailed process of PCAT segmentation was displayed in the Supplementary Materials.

In total, we extracted 107 radiomics features (Supplementary Materials) from PCAT surrounding plaques with the reference of the image biomarker standardization initiative (IBSI) [26] using an artificial intelligence kit (A.K., GE Healthcare).

**Fig. 2:**
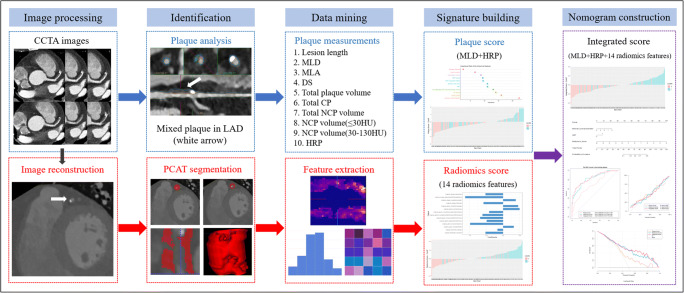
A flow chart displaying the process for development of radiomics-based integrated score. CCTA, coronary computed tomography angiography; PCAT, pericoronary adipose tissue; LAD, left anterior descending; HRP, high-risk plaque; MLD, minimal lumen diameter; MLA, minimal lumen area; DS, diameter stenosis; CP, calcified plaque; NCP, non-calcified plaque

In order to ensure the robustness and stability of our built models, intra-class correlation coefficient (ICC) was used to evaluate the inter-reader reproducibility of radiomics features (Supplementary Materials). Features with an ICC value > 0.75 were considered a good agreement [27] and remained for subsequent analysis. Figure 2 shows the radiomics workflow of this study.

### Feature selection and prediction model building

We developed three predictive models to determine the discrimination of patients with subsequent ACS; their building process was detailed as follows:

#### Plaque score

Univariate logistic regression was used to select the plaque predictors with *p* < 0.05, then multivariate logistic regression was used to identify the significant features using the backward stepwise elimination method. Finally, a plaque score was calculated based on the above-selected plaque predictors weighted by their respective coefficients.

#### Radiomics score

Firstly, we selected features with an ICC > 0.75 for subsequent analysis. Secondly, to reduce the risk of overfitting, we further eliminated features with highly pairwise correlations at the level of |*r*|≥ 0.9. Thirdly, the least absolute shrinkage and selection operator (LASSO) regression was conducted to select the most significant radiomics features with non-zero coefficient using 10-fold cross-validation (Supplementary Materials). Then radiomics score was calculated for each patient via a linear combination of the selected features which weighed by their respective coefficient.

#### Integrated score

Using multivariate logistic regression, an integrated score was calculated for each patient via a linear combination of radiomics score and the selected plaque predictors.

### Statistical analysis

All statistical analyses were performed with R software (version 3.5.1; http://www.Rproject.org). R packages used in this study were listed in the Supplementary Materials. Continuous variables were presented as mean S ± SD or median (25th, 75th percentile) according to the data distribution. The chi-square test was used to compare categorical variables between two groups; either Student’s *t*-test or Mann-Whitney *U* test was used for the continuous variables as appropriate.

The performance of three developed models was evaluated with discrimination, calibration, and clinical application in an independent test dataset.

#### Discrimination

The receiver operating characteristic (ROC) curve was used to evaluate the diagnostic performance of three predictive models in identifying patients with subsequent ACS. The DeLong test was used to compare the area under ROC curves (AUC) between different models or different datasets.

#### Calibration

Calibration curves were plotted to assess the agreement between the observed outcome frequencies and predicted probabilities of three predictive models. The Hosmer–Lemeshow test was used to determine the goodness of fit of the models, and *p* > 0.05 was considered good calibration.

#### Clinical application

Decision curve analysis (DCA) was conducted to evaluate the clinical usefulness of three predictive models by quantifying the net benefits at different threshold probabilities.

Stratification analyses were performed on different CT protocols and different scanning sites.

## Results

### Patients’ clinical characteristics

Patients’ clinical characteristics in the training and test dataset were detailed in the Supplementary Materials. Of patients with ACS, 13 (14.44%) presented with ST segment elevation myocardial infarction (STEMI), 25 (27.78%) presented with non-ST segment elevation myocardial infarction (NSTEMI), and 52 (57.78%) with unstable angina (UA). The average duration time between coronary CTA scan and the occurrence of ACS was 15.19 ± 11.15 months. There were no significant differences between the two groups with regard to the distribution of clinical characteristics in both the training and test dataset.

### Coronary plaque analysis

Conventional plaque characteristics in the training and test dataset are presented in Table 1. Of the plaque analysis, we observed that 4 plaque features (consisting of minimal lumen diameter (MLD), minimal lumen area(MLA), DS, and HRP) were significantly different between the two groups in the training dataset, but no statistical significance was found in the test dataset. There was no significant difference in all plaque features between the training and test dataset (*p* > 0.05).

**Table 1:** Plaque characteristics of the study population and three predictive model score in the training and test dataset

Characteristics	Training dataset			Test dataset			*p* valu**e**
	Control (*n* = 67)	ACS (*n* = 67)	*p* value	Control (*n* = 23)	ACS (*n* = 23)	*p* value	
Plaque characteristics							
Lesion distribution			0.116			0.280	0.301
RCA	13 (19.40)	14 (20.90)		4 (17.39)	8 (34.78)		
LAD	49 (73.13)	40 (59.70)		18 (78.26)	14 (60.87)		
LCX	5 (7.46)	9 (13.43)		1 (4.35)	0 (0.00)		
D	0 (0.00)	4 (5.97)		0 (0.00)	1 (4.35)		
LL, median (25%, 75%) mm	14.20 (6.84, 22.14)	13.70 (8.62, 22.76)	0.342	17.80 (10.68, 30.30)	17.60 (6.44, 33.26)	0.742	0.140
MLD, median (25%, 75%) mm	1.20 (0.80, 1.88)	0.90 (0.42, 1.30)	0.001*	1.14 ± 0.58	0.91 ± 0.60	0.191	0.559
MLA, median (25%, 75%) mm^2^	1.60 (0.82, 3.34)	1.00 (0.52, 2.00)	0.013*	1.10 (0.82, 2.10)	1.10 (0.56, 2.10)	0.475	0.610
Diameter stenosis			0.022*			0.240	0.972
Mild (< 50%)	27 (40.30)	17 (25.37)		9 (39.13)	5 (21.74)		
Moderate (50 to 70%)	26 (38.81)	25 (37.31)		9 (39.13)	11 (47.83)		
Severe (≥ 70%)	14 (20.90)	25 (37.31)		5 (21.74)	7 (30.43)		
Plaque volume, median (25%, 75%) mm^3^							
Total plaque volume	48.30 (20.48, 73.24)	44.30 (24.68, 79.72)	0.915	35.30 (25.62, 115.86)	44.7 (20.12, 102.36)	0.660	0.871
CP volume	32.90 (14.96, 53.78)	30.20 (15.12, 51.66)	0.836	25.30 (9.38, 107.28)	37.60 (13.10, 80.96)	0.965	0.698
NCP volume	5.60 (1.54, 18.46)	8.90 (2.10, 23.28)	0.390	8.60 (1.26, 21.52)	5.00 (1.30, 31.42)	0.956	0.732
Low-attenuation NCP (≤ 30 HU)	0.50 (0.00, 1.68)	0.80 (0.00, 2.88)	0.214	0.60 (0.00, 1.88)	0.90 (0.00, 3.26)	0.590	0.914
NCP (30–130 HU)	5.20 (1.40, 16.98)	5.40 (1.72, 18.96)	0.519	7.60 (1.22, 16.62)	4.60 (1.22, 24.00)	0.982	0.748
High-risk plaque, *n* (%)	5 (7.46)	17 (25.37)	0.005*	3 (13.04)	8 (34.78)	0.084	0.257
Plaque score (model 1)	−0.19 (−0.83, 0.18)	0.28 (−0.35, 0.74)	< 0.001*	−0.05 ± 0.66	0.45 ± 0.93	0.042*	0.195
Radiomics score (model 2)	−0.90 ± 1.33	0.94 ± 1.47	< 0.001*	-0.85 ± 1.17	0.69 ± 1.61	0.001*	0.725
Integrated score (model 3)	−1.00 ± 1.36	1.03 ± 1.52	< 0.001*	−0.81 ± 1.10	0.88 ± 1.56	< 0.001*	0.934

*LAD*, left anterior descending; *RCA*, right coronary artery; *LCX*, left circumflex; *D*, diagonal branch; *LL*, lesion length; *MLD*, minimal lumen diameter; *MLA*, minimal lumen area; *CP*, calcified plaque; *NCP*, non-calcified plaque. High-risk plaque was defined as the presence of at least two of the adverse plaque characteristics including low-attenuation plaque, positive remodeling, spotty calcification, and napkin-ring sign

*p* values were derived from the univariable association analysis between different variables, * data are means with a statistical difference. *p* value reflected the differences between the training and test dataset

### Feature selection and prediction model building

Three predictive models were respectively developed to determine the predictive capability of future ACS. The detailed calculation formula and the distribution of three models are shown in Supplementary Materials.

#### Plaque score

Among conventional plaque characteristics, we found that two plaque features (MLD and HRP) were significantly associated with the occurrence of future ACS using univariate and multivariate logistic regression, then they were combined to construct a plaque score by multivariate logistic regression analysis.

#### Radiomics score

A total of 103 radiomics features showed good stability with an ICC > 0.75 on inter-observer analysis(Supplementary Materials); after redundancy with spearman correlation analysis, 41 features remained; finally, 14 significant radiomics features with none-zero coefficient were selected after LASSO regression analysis, which was devoted to calculating radiomics score (Supplementary Materials).

#### Integrated score

**Fig. 3:**
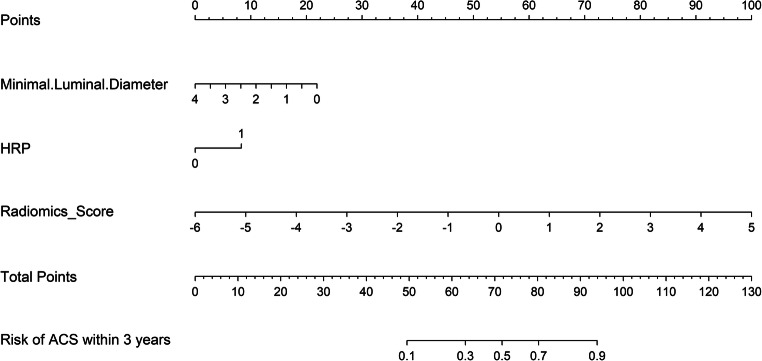
Developed integrated model nomogram. The integrated score nomogram was developed in the training dataset with minimal luminal diameter (MLD), high-risk plaque (HRP), and a Rad-score of the selected radiomics features incorporated

An integrated score incorporating selected plaque predictors (MLD, HRP) with radiomics score were further developed for predicting subsequent ACS, and we presented it as a nomogram (Fig. 3).

### Performance evaluation

#### Discrimination

**Fig. 4:**
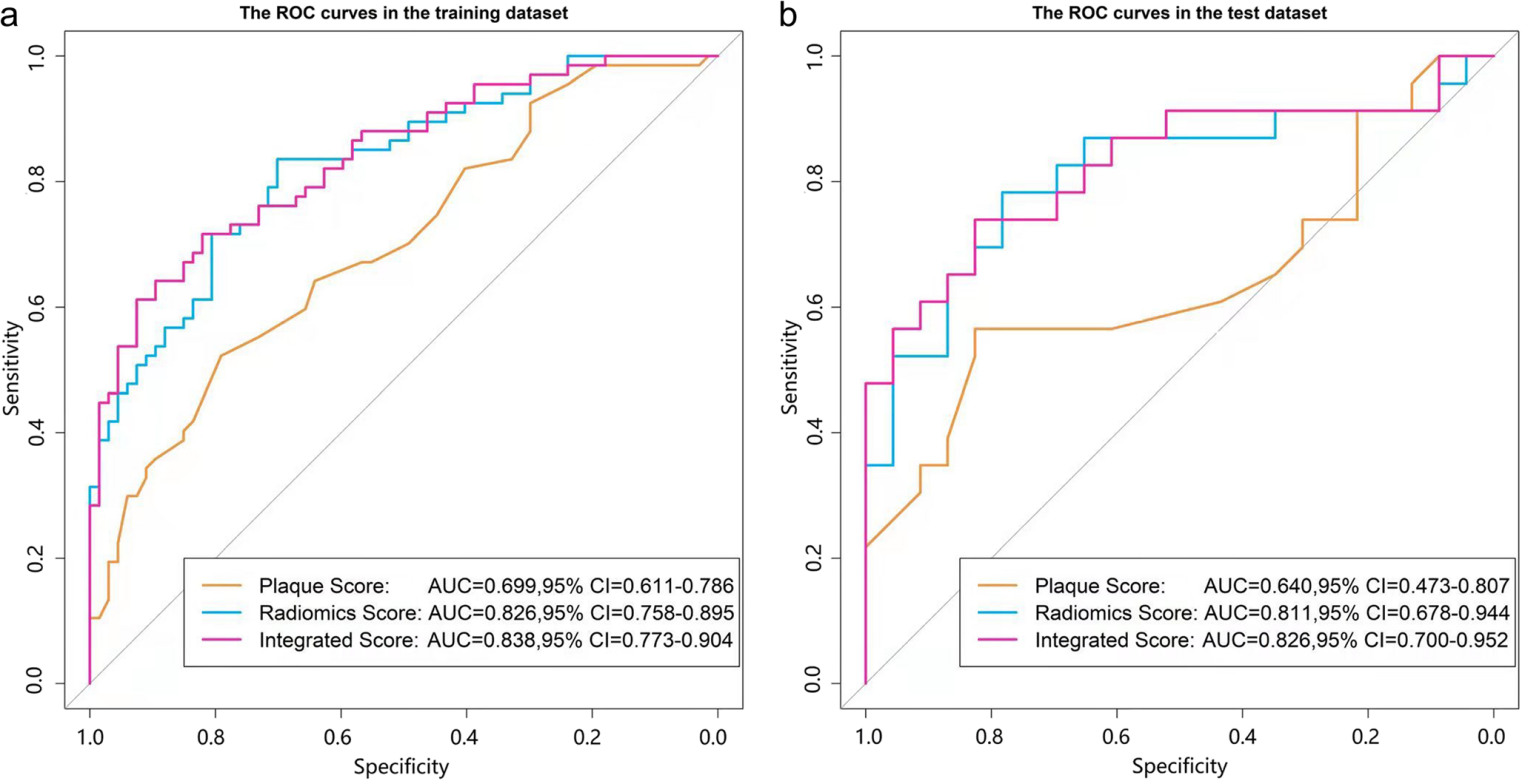
Comparison of ROC curves for the plaque score (yellow lines), radiomics score(blue lines) and integrated score(pink lines) in the training (**a**) and test (**b**) dataset

ROC curves of radiomics score, plaque score, and integrated score were plotted to reveal the performance of discriminating ACS in the training and test dataset (Fig. 4). The values of AUC, sensitivity, and specificity were measured to quantify the discrimination ability of three predictive models (Table 2). The radiomics score achieved superior discrimination in the training and test dataset (AUC = 0.826 [95%CI: 0.758–0.895], 0.811 [95%CI: 0.678–0.944]) compared with plaque score (AUC = 0.699 [95%CI: 0.611–0.786]), 0.640 [95%CI: 0.473–0.807]), while the improvement of integrated score discriminating capability (AUC = 0.838 [95% CI: 0.773, 0.904], 0.826 [95% CI: 0.700, 0.952]) was non-significant compared with radiomics score.

**Table 2:** Comparison of AUCs between the plaque score, radiomics score, and integrated score

Model		Training dataset (*n* = 134)			Test dataset (*n* = 46)			*p* value (Delong test)
	Cutoff	AUC (95% CI)	SPE	SEN	AUC (95% CI)	SPE	SEN	
Plaque score	0.230	0.699 (0.611–0.786)	0.791	0.522	0.640 (0.473–0.807)	0.739	0.565	0.544
Radiomics score	−0.293	0.826 (0.758–0.895)	0.701	0.836	0.811 (0.678–0.944)	0.696	0.826	0.841
Integrated score	0.201	0.838 (0.773–0.904)	0.821	0.716	0.826 (0.700–0.952)	0.826	0.696	0.865

*AUC*, area under ROC curve; *95% CI*, 95% confidence interval; *SEN*, sensitivity; *SPE*, specificity. *p* value reflects the differences of three predictive models between the training and test dataset respectively

Delong test revealed that there was no statistical difference of the AUCs of three models between the training and test dataset, with *p* values of 0.544, 0.841, and 0.865, respectively (Table 2). Furthermore, there was no significant difference between the radiomics score and integrated score in the training dataset (*p* = 0.314), but both of them are statistically superior to plaque score (*p* = 0.009 and *p* < 0.001, respectively); there was no statistical difference between three scores in the test dataset (all *p* > 0.05).

#### Calibration

**Fig. 5:**
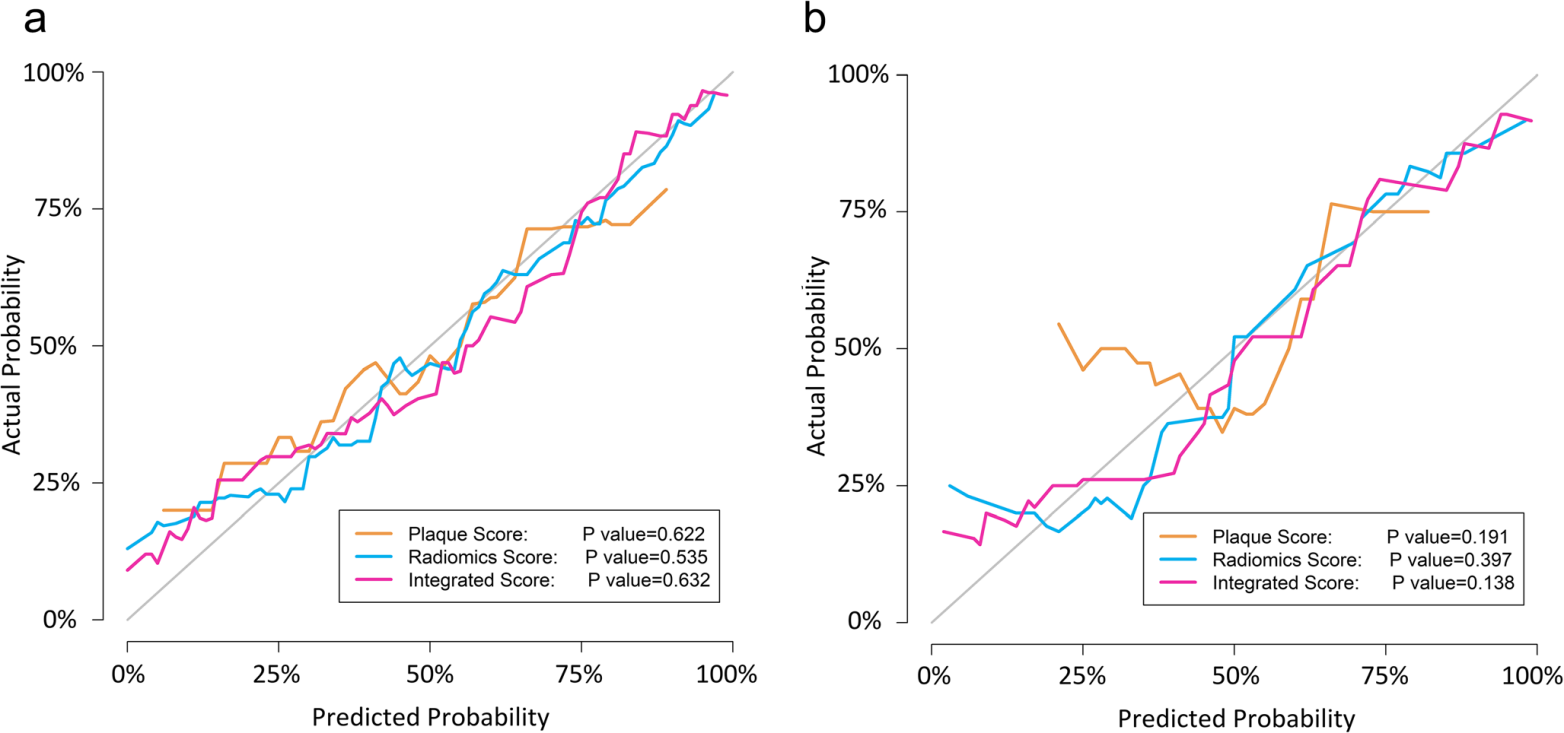
Calibration curves for the integrated score (pink lines), radiomics score (blue lines), and plaque score (yellow lines) in the training (**a**) and test (**b**) dataset. The calibration curves represented the fitness of three models between the predicted probability and the real outcomes. A closer fitness to the diagonal line represented a well-calibrated model. The fitness of integrated score and radiomics score is superior to plaque score in the test dataset since calibration curves of the former are closer to the diagonal line

The calibration curves of three predictive models all demonstrated a good fitness (*p* > 0.05 in the Hosmer–Lemeshow test) between prediction and observation for the probability of ACS in both training and test datasets (Fig. 5).

#### Clinical application

**Fig. 6:**
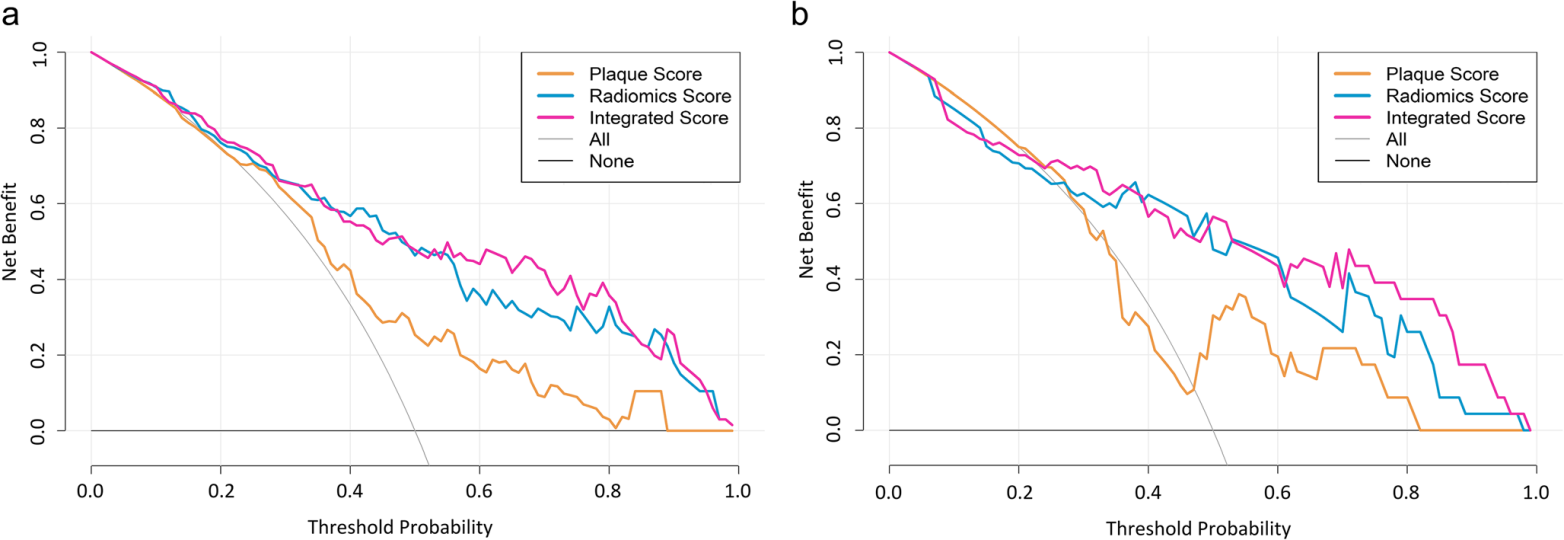
Decision curve analysis for the integrated score (pink lines), radiomics score (blue lines), and plaque score (yellow lines) in the training (**a**) and test (**b**) dataset. The *y*-axis represented the net benefit, the *x*-axis represented threshold probability. The gray curve line represented the assumption that all patients have an ACS event, while the black curve line represented the assumption that no patients have ACS. The pink line, blue line, and yellow line represented the net benefit of the integrated score, the radiomics score, and plaque score, respectively. The integrated score and radiomics score had a higher net benefit than a plaque score

The decision curves displayed the clinical usefulness of three predictive models by comparing the net benefits at different threshold probabilities in the training and test dataset and demonstrated that the integrated score and radiomics score had a higher net benefit than plaque score (Fig. 6).

#### Stratified analysis

As shown in Supplementary Materials, stratified analysis revealed that the performance of three predictive models was not affected by tube voltage (100kv, 120kv), CT version (iCT, IQon CT), different sites of our hospital (Nanhu site, Huaxiang site) (all *p* > 0.05).

## Discussion

In this study, we developed an integrated score that incorporated radiomics features of PCAT surrounding target lesions and significant plaque predictors based on CCTA and validated the performance with respect to discrimination, calibration, and clinical application. This integrated score displayed superior diagnostic performance in the prediction of future ACS within 3 years compared with plaque score.

As many ACS events usually occur in patients without obstructive plaques, we should pay more attention to the identification of vulnerable plaques instead of the degree of luminal stenosis. The current CCTA could not only identify obstructive atherosclerotic plaques and plaque burden but also evaluate HRP features beneficial to risk stratification [4, 28]. Although the detection of HRP features has provided the incremental predictive value of coronary events to a certain extent [29], they are not a direct indicator of inflammation, just anatomical signs of vulnerable plaques and markers of the risk of rupture [4]. Recent studies [10] have suggested that coronary inflammation drives dynamic changes in perivascular adipose tissue (PVAT) composition, captured by a novel CCTA-derived imaging biomarker, the perivascular FAI, which reflects inflammation-induced PVAT changes in adipocyte size and lipid content are related to CT attenuation gradients. However, this metric based on CT attenuation simply reveals the average voxel intensity values without considering the complex spatial relationship among voxels. Radiomics enables help to provide the spatial distribution of voxel gray-level intensities and a quantification of heterogeneity [16].

In our study, we selected 14 most significant predictors from 107 radiomics features of PCAT based on CCTA using multivariate logistic regression analysis and found that patients of which PCAT with lower uniformity and higher heterogeneity were correlated with high possibility of future ACS, which indicated that heterogeneity of PCAT revealed by radiomics may reflect early pathophysiological changes in the adipose tissue around plaques; besides, the difference of PCAT radiomics parameters between two groups may be influenced by the related local inflammatory response, and PCAT radiomics characterization may be closely associated with subsequent plaque rupture. We further developed and validated that integrated score combining radiomics features with significant plaque predictors yielded a good diagnostic performance in identifying patients with ACS in both training and test datasets. Indeed, a recent prospective case-control study [23] suggested that a distinct radiomics phenotype of PCAT exists between patients with acute MI and patients with stable or no CAD, especially textural and geometric features that provided more additional information than the average attenuation of PCAT; moreover, their radiomics-based model outperformed PCAT attenuation-based model in identifying patients with MI. Oikonomou EK et al [22] developed a novel fat radiomics profile (FRP) using CCTA-based radiomics phenotyping of coronary PVAT and validated its performance in three different patient cohorts, which show that FRP contributed to further improve cardiac risk prediction for major adverse cardiac events beyond common established risk factors. Nevertheless, our study showed that the selected plaque predictors could not significantly improve the discriminating ability of integrated score in identifying patients with subsequent ACS compared with radiomics score, which could not mean that we did not need conventional plaque characteristics anymore to determine future ACS risk in the light of small sample size.

Based on our results, there was a statistical difference in MLD, MLA, DS, and HRP of plaque characteristics between two groups in the training dataset, but none of them was significantly different in the test dataset, which may be affected by the small sample size. Simultaneously, HRP was a more significant predictor among them. The present study [3] based on CCTA also showed that HRP was an independent predictor of ACS. Lee JM et al [30] also displayed that the culprit lesions had more frequent HRP than nonculprit lesions, and HRP further improved discriminatory ability in the identification of subsequent ACS. Nonetheless, our plaque score that was derived from MLD and HRP did not show a much better performance on predicting ACS outcomes in the test dataset. This might be due to the occurrence of ACS events is affected by many factors. Although coronary CTA has the ability to identify HRP features, there are still certain limitations in detecting small but vulnerable plaques which are potential to either rupture or rapidly progress to obstructive heart disease [31]. Moreover, HRP usually has a heterogeneous natural history and only a small proportion of them actually cause ACS events [32], several pieces of evidence suggested that half of ACS events happened without anatomically significant atherosclerotic plaques. Motoyama S et al showed that 83.7% of HRP identified by CCTA did not cause any ACS events [3].

Application of CCTA-based radiomics analysis to PCAT surrounding lesions in patients with ACS may increase our understanding of the related inflammatory response in the pericoronary environment. We developed a more comprehensive ML model for identifying patients with future ACS at a noninvasive imaging level by integrating radiomics features with plaque predictors, then found that the integrated score obviously added incremental discriminatory value in identifying patients with subsequent ACS over plaque score. The better performance of the radiomics-based score demonstrated that radiomics methods could extract more predictive information from PCAT based on CCTA than conventional plaque characteristics and have the potential to enhance the predictive ability of subsequent ACS. These findings further proved the important role of radiomics information in PCAT for the prediction of ACS.

There are still several limitations in our study. Firstly, we presented a retrospective case-control study design within a single center. The sample size of our work is relatively small; there is still a need for further external validation in an independent cohort to verify our findings. Secondly, although our work performed stratified analysis on three predictive models that indicated a good reproducibility and robustness exist, the application of our predictive models to general populations is limited to a single-center study. Several studies [33–35] indicated that image acquisition, reconstruction, and analysis have a certain impact on the reproducibility of radiomics features. Thirdly, our study merely concentrated on PCAT radiomics phenotyping at a per-patient level, further study extended to a larger population at per-lesion level would be carried out. Furthermore, the current method of manually delineating ROI in our study is relatively time-consuming and easy to be affected by human factors, especially the process of image reconstruction greatly affected by the experience of radiologists. Although we did ICC to verify the stability of the extracted features, eliminate unstable features and try to build a robust model, we still hope to use semi-automatic or fully automatic segmentation technology to perfect this part of the work.

In conclusion, CCTA-based radiomics signature of PCAT could have the potential to improve the predictive ability of subsequent ACS. Radiomics-based integrated score significantly outperformed plaque score in identifying future ACS within 3 years.

## Supplementary information

ESM 1 (DOCX 5574 kb)

## Abbreviations

ACS: Acute coronary syndrome
APC: Adverse plaque characteristics
AUC: Area under the curve
CAD: Coronary artery disease
CCTA: Coronary computed tomography angiography
CP: Calcified plaque
DCA: Decision curve analysis
FAI: Fat attenuation index
HRP: High-risk plaque
IBSI: Image biomarker standardization initiative
ICC: Intra-class correlation coefficient
LASSO: least absolute shrinkage and selection operator
ML: Machine learning
MLA: Minimal lumen area
MLD: Minimal lumen diameter
NCP: Noncalcified plaque
NSTEMI: Non-ST-segment elevation myocardial infarction
PCAT: Pericoronary adipose tissue
PVAT: Perivascular adipose tissue
ROC: Receiver operating characteristic
ROI: Region of interest
STEMI: ST-segment elevation myocardial infarction
UA: Unstable angina

## Declarations

### Guarantor

The scientific guarantor of this publication is Yang Hou.

### Statistics and biometry

Yan Guo kindly provided statistical advice for this manuscript.

### Methodology

• Retrospective

• Case-control study

• Performed at one institution
